# Maternal depression around pregnancy and risk of mental disorder among offspring: a population-based cohort study with sibling comparison

**DOI:** 10.1192/j.eurpsy.2025.360

**Published:** 2025-08-26

**Authors:** A. Nevriana, D. Lu

**Affiliations:** 1 Institute of Environmental Medicine, Karolinska Institutet, Stockholm, Sweden

## Abstract

**Introduction:**

Previous studies have indicated a potential increase in risks of mental disorders among the offspring of mothers with depressive disorders. However, the association between maternal exposure at different developmental periods, including the period around pregnancy, and the offspring’s risk for adverse mental health outcomes remain unclear. Additionally, there is a lack of studies controlling for potential confounding by familial factors.

**Objectives:**

To determine the association between the timing of maternal depression around pregnancy and the risk of mental disorders among the offspring.

**Methods:**

Population-based cohort study using linkage from Swedish national registers including 4,051,192 live singleton births in 1973-2010. Individuals were followed from age 3 until the first date of mental disorders diagnosis, emigration, death or 31 December 2013. We included the following diagnoses: depression, postpartum depression, neurotic disorders, stress-related disorders, alcohol use disorders, drug use disorders, attention deficit hyperactivity disorder (ADHD), autism spectrum disorder (ASD), intellectual disability, behavioural disorders (child-/adolescent-onset), schizophrenia, other psychotic disorders, bipolar disorders, eating disorders, personality disorders, and others. Timing of maternal depression was defined as the earliest date of diagnosis or antidepressant dispensation around pregnancy, categorised into ‘1 year before conception’, ‘during pregnancy’, and ‘1 year after childbirth.’ Hazard ratios (HR) were estimated using Cox regression, adjusting for potential confounders. We also stratified the associations by age at follow up and birth year. To account for familial confounding, comparison was also made within full siblings.

**Results:**

In the population-based analysis, maternal depression was associated with a higher risk of overall mental disorder diagnosis in offspring in all three time periods, although the association tends to be stronger during the first year before conception (HR 1.90, 95% CI 1.78-2.03) and somewhat attenuated afterwards (HR during pregnancy 1.77, 95% CI 1.69-1.84; HR 1 year after childbirth 1.68, 95% CI 1.57-1.79; Figure 1). However, the associations were attenuated to null in the sibling analysis (HR overall mental disorders 1 year before conception 0.94, 95% CI 0.83-1.07; during pregnancy 1.08, 95% CI 0.98-1.17; 1 year after childbirth 1.00, 95% CI 0.88-1.13). Similar patterns were observed in most mental disorder diagnoses (Figure 1), across age at diagnosis (Figure 2), and birth year (Figure 3).

**Image 1:**

**Figure:**
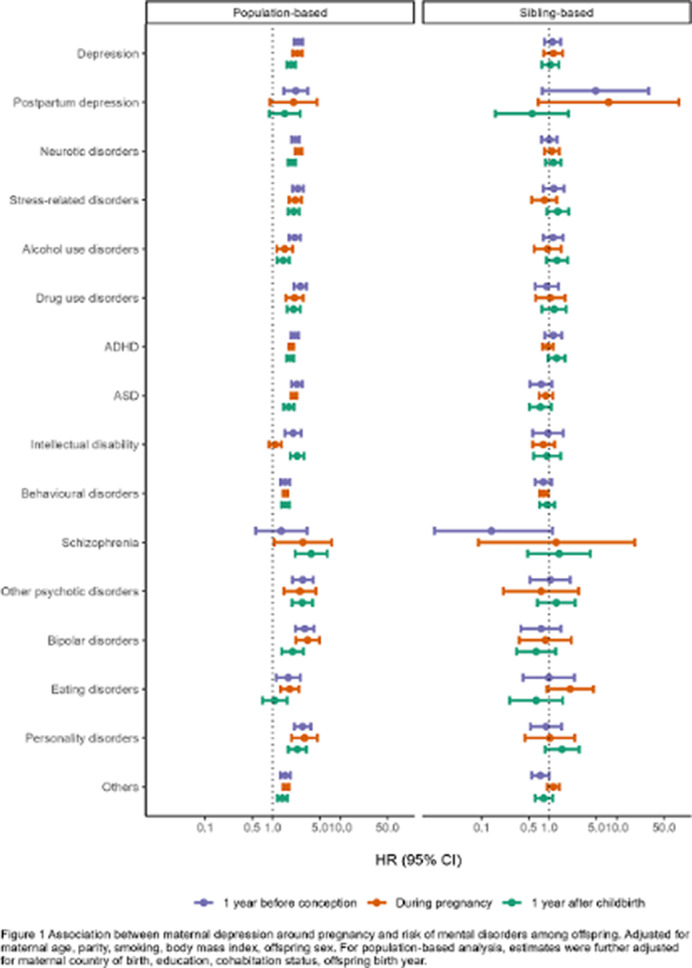

**Image 2:**

**Figure:**
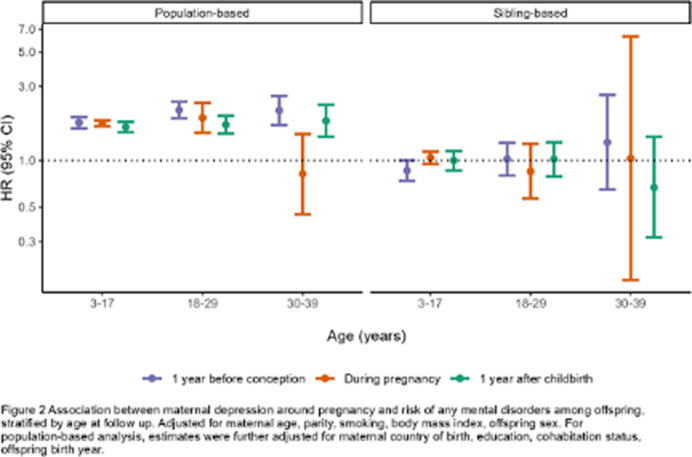

**Image 3:**

**Figure:**
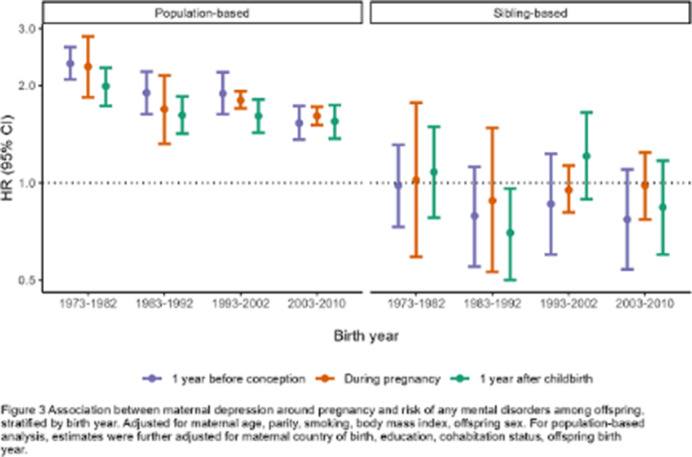

**Conclusions:**

While maternal depression before, during, and after pregnancy is predictive for the offspring’s mental health development, the link is likely driven by shared familial genetic and environmental factors.

**Disclosure of Interest:**

None Declared

